# The Efficacy of Topical Cosmetic Containing Alpha‐Arbutin 5% and Kojic Acid 2% Compared With Triple Combination Cream for the Treatment of Melasma: A Split‐Face, Evaluator‐Blinded Randomized Pilot Study

**DOI:** 10.1111/jocd.16562

**Published:** 2024-11-18

**Authors:** Pimpa Tantanasrigul, Apinya Sripha, Bunchai Chongmelaxme

**Affiliations:** ^1^ Department of Medical Services, Ministry of Public Health Institute of Dermatology Bangkok Thailand; ^2^ Department of Social and Administrative Pharmacy, Faculty of Pharmaceutical Sciences Chulalongkorn University Bangkok Thailand

**Keywords:** alpha‐arbutin 5%, efficacy, kojic acid 2%, melasma, safety, triple combination cream

## Abstract

**Background:**

While the gold standard treatment for melasma is triple combination cream (TCC), arbutin and kojic acid demonstrate their benefits and may be used as an alternative.

**Aims:**

To investigate the efficacy of cream containing alpha‐arbutin 5% and kojic acid 2% (AAK) compared with TCC for melasma treatment.

**Patients/Methods:**

A split‐faced, randomized study was conducted among 30 participants with melasma, and all were randomized to receive AAK or TCC on each side of their face for 12‐week along with 4‐week follow‐up period. The melanin index (MI), modified Melasma Area Severity Index (mMASI), and physician global assessment (PGA) scores were used to measure the effectiveness of interventions. Recurrence of melasma after treatment discontinuation was evaluated by MI and mMASI. Patient satisfactions and adverse effects were also evaluated. In the analysis, the mean difference (MD) was used for MI and mMASI, while Wilcoxon signed‐rank test was for the PGA scores, adverse effects, and patient satisfaction.

**Results:**

The MD of MI and mMASI scores were not different between groups (mMASI [*p* = 0.344] and MI [*p* = 0.268]). The PGA scores only showed improvement on the TCC‐treated side (*p* = 0.032). Compared to the AKK group, the subjects with TCC showed higher severity of recurrence (MI [*p* = 0.004] and mMASI [*p* = 0.045]). No difference in patient satisfaction score between the groups, but erythema and stinging were higher in the TCC group.

**Conclusions:**

The AAK cream appeared to be effective for melasma treatment, highlighting a lower recurrent rate and fewer adverse events than standard therapy.

**Trial Registration:** thaiclinicaltrials.org: TCTR20230124004

## 1 Introduction

Melasma is a chronic hyperpigmented skin disorder that usually distributes symmetrically on the face. Patients with Fitzpatrick skin phototypes III and IV appeared to be the most affected population [1]. The etiopathogenesis of melasma is not fully elucidated. Melanocytes activation and proliferation and complex interplays of cytokines lead to the development of melasma. Exposure to ultraviolet radiation is the leading cause of melisma; however, there are other several aggravating factors such as genetic predisposition, hormonal changes, and certain medications [2].

The current gold standard treatment of melasma is triple combination cream (TCC), approved by the US Food and Drug Administration (FDA) as Tri‐Luma, which contains hydroquinone 4%, tretinoin 0.05%, and fluocinolone acetonide 0.01%. The efficacy of TCC had been delineated in many studies as an effective anti‐melasma agent [3, 4, 5, 6]. First, hydroquinone is a hydroxyphenolic compound that acts as a tyrosinase inhibitor by preventing conversion of DOPA into melanin, enhance degradation of melanosome, and decrease melanocytes DNA synthesis [3, 4]. Second, tretinoin inhibits melanin synthesis and transcription of tyrosinase enzyme [5]. It also exhibits an anti‐inflammatory property and enhances keratinocytes turnover thus reduces melanosome transfer. Third, a low‐potency fluorinated corticosteroid, for example, fluocinolone acetonide, inhibits prostaglandins and other inflammatory cytokines that activate melanin synthesis by melanocytes [5]. Furthermore, corticosteroids also alleviate irritation caused by hydroquinone and tretinoin [6, 7]. Regardless of the beneficial effects of TCC, persistent use of corticosteroids in melasma raised a concern for the increased risk of skin atrophy and hypopigmentation. In addition, long‐term application of hydroquinone is associated with the development of exogenous ochronosis which is a permanent accumulation of homogentisic acid in the skin [4, 7].

Natural compounds such as arbutin and kojic acid can be used to treat melasma; arbutin, extracted from bearberry leaves, is a hydroquinone derivative. There are alpha and beta isomers, the latter is recognized as arbutin while the former, alpha‐arbutin is superior to arbutin in inhibiting tyrosinase enzyme [8, 9]. Alpha‐arbutin interrupts melanin synthesis by interfering with tyrosinase activity as well as impeding melanosome maturation [10, 11]. In addition, kojic acid is a metabolite of *Aspergillus* and *Penicillium* spp. [7] It chelates with copper which is the substrate of tyrosinase therefore inhibits this enzyme [6, 11]. Scavenging of free radicals by kojic acid further enhance its lightening effect [12]. Combination of kojic acid with other anti‐melasma compounds is recommended since the treatment outcomes were better than kojic acid monotherapy [10].

The previous study by Arruda et al. [9] revealed that cosmetic cream containing arbutin 5% and kojic acid 2% was comparable to hydroquinone 4% in decreasing melasma evaluated by modified Melasma Area Severity Index (mMASI) scale. To date, clinical evidence investigating the effects of arbutin and kojic acid among Thai patients with melasma is limited. As unfavorable adverse outcomes may occur following the TCC application, natural compounds have become more attractive as alternative treatment. This study aims to compare the efficacy of topical cream containing alpha‐arbutin 5% and kojic acid 2% (AAK) with TCC for melasma treatment among Thai population. In addition, patient satisfaction and adverse effects of interventions were also evaluated. The findings of this are expected to provide healthcare professionals with supportive evidence of the AAK for the treatment of melasma.

## 2 Materials and Methods

### 2.1 Study Design

**FIGURE 1:**
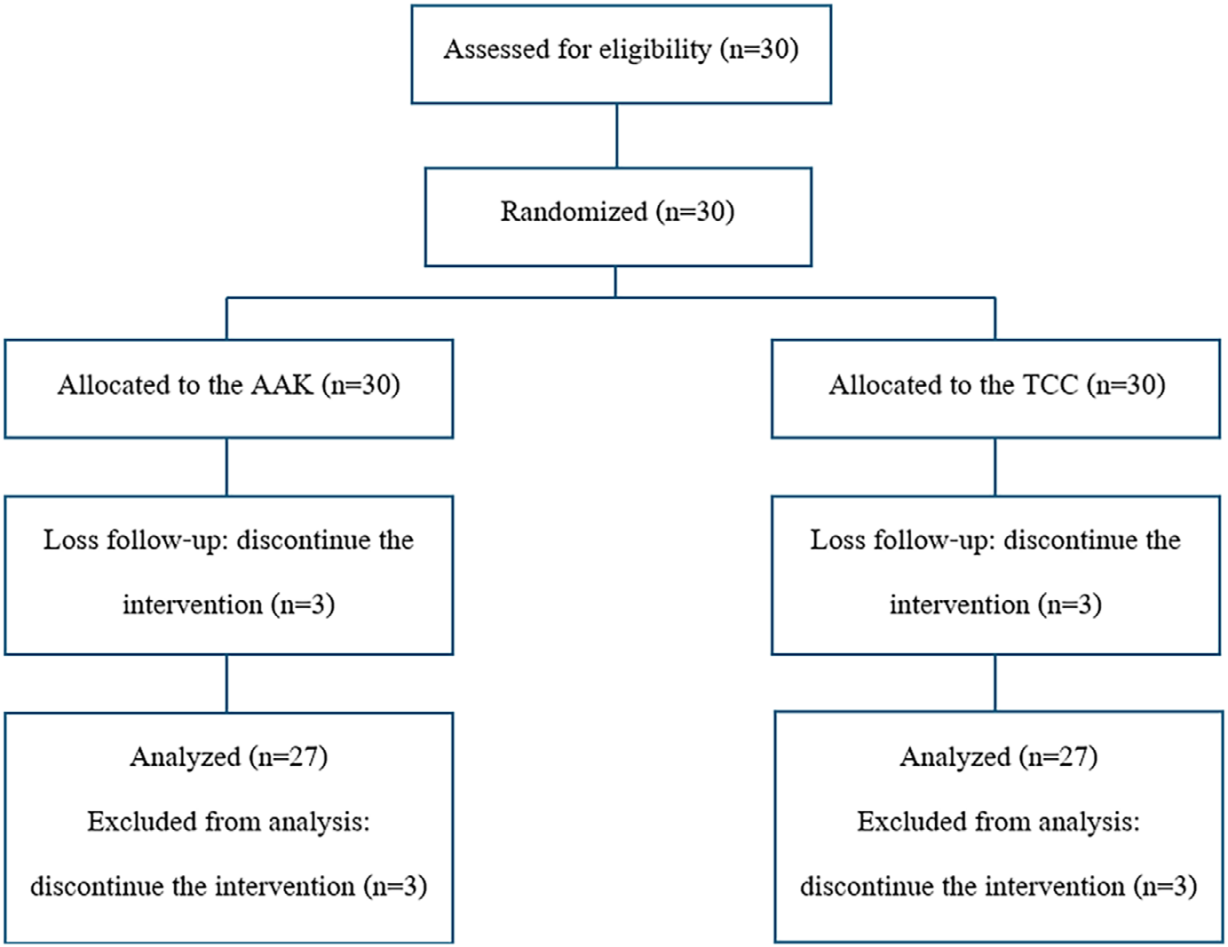
Study enrollment flowchart and sample retention. AAK, alpha‐arbutin 5% and kojic acid 2%; TCC, triple combination cream.

A split‐faced, randomized pilot study was conducted at the Institute of Dermatology Thailand from May to December 2022. The study protocol was approved by the ethical committee and all subjects gave consent prior to enrollment. Thirty subjects were recruited, and 27 subjects completed the study **(**Figure 1 **)**. Eligible participants were adults aged between 25 and 60 years with bilateral and symmetrical facial melasma. Exclusion criteria were those with the history of allergic to ingredients in the TCC and AAK; current treatment of topical and/or oral medicines that had anti‐melasma effects; aesthetic treatments of melasma within 3 months prior to recruitment. Pregnancy and lactation were also excluded.

All participants were randomized by simple randomization to apply cream containing the AAK on one side of their face (twice daily) and the TCC on another side (once daily before bedtime). The participants received instructions to use broad‐spectrum sunscreen SPF 50 and to avoid sun exposure from 9 a.m. until 5 p.m., and all had to refrain from using any treatment and cosmetics that could alter pigmentation throughout the study. The duration of treatment was 12 weeks followed by a 4‐week period after treatment discontinuation.

### 2.2 Photographic Documentation and Clinical Assessment

Photography was taken at baseline and during follow‐up visits. Canfield VISIA‐CR system (Canfield Scientific, Parsippany, NJ) was utilized capturing frontal, left lateral 37°, and right lateral 37° views. Clinical evaluation was performed at Week 4, 8, 12, and 16. The Melanin index (MI) was used to evaluate the severity of pigmentation measured by a narrow‐band reflectance spectrophotometer (Mexameter MX18; Courage + Khazaka electrical GmbH). At each visit, three blinded dermatologists assessed clinical photographs, and scored the mMASI and the physician global assessment (PGA). The mMASI scoring system calculated the darkness and area of melasma, while the PGA scores was graded according to the improvement of hyperpigmentation; the scale score ranged from 0 to 4 (0 = no improvement, 1 = mild [1–25%], 2 = moderate [26–50%], 3 = good [51–75%], 4 = excellent [>75%]).

### 2.3 Patient Satisfaction and Adverse Outcomes Assessment

The levels of patient satisfaction were rated by participants as follows: very satisfied, satisfied, fair, and dissatisfied, and the side effects were assessed by dermatologists on a 4‐point scale: 0 = none, 1 = mild, 2 = moderate, 3 = severe.

### 2.4 Statistical Analysis

Statistical analysis was performed using Stata version 17. Intraclass correlation coefficients of three blinded dermatologists were calculated to assess intra‐rater reliability. The mean difference (MD) of MI and mMASI scores between two groups were determined by using a mixed‐model analysis, while the PGA scores, patient satisfaction, and side effects were compared by using Wilcoxon Signed Ranks test. All the statistical significance were defined as *p*<0.05.

## 3 Results

Of the 30 participants, three refused to continue the interventions, and none was lost due to adverse reactions. As such, 27 were included in the intention‐to‐treat analysis. All participants had bilateral melasma, and most of them were female (85.2%) with mean age approximately 46 years. Their types of melasma and severity levels were described in Table 1.

**TABLE 1:** Demographics characteristics.

Characteristic	Number (%)
Female	23 (85.2%)
Male	4 (14.8%)
Age (year)	46.59 ± 7.29
Fitzpatrick skin type	
III	13 (48.1%)
IV	13 (48.1%)
V	1 (3.7%)
Type of melasma	
Epidermal	7 (25.9%)
Dermal	10 (37%)
Mixed	10 (37%)
Melasma severity scale	
Mild	11 (40.7%)
Moderate	10 (37%)
Severe	6 (22.2%)

The MI scores at baseline were 286.89 ± 70.83 for the AAK groups, and 283.36 ± 73.97 for the TCC group, while that at Week 12 were 266.77 ± 67.75, and 241.68 ± 73.26, for the TCC and AAK, respectively. The MD of MI scores between those groups at Week 12 was 21.64 (95% confidence interval [CI]: −1.70, 44.99) (*p* = 0.069). Likewise, at the follow‐up period Week 16, nonsignificant difference of the MD was shown between the groups (*p* = 0.268) (Table 2). The mMASI scores demonstrated similar results as MI. The mean mMASI scores at Week 12 were 2.19 ± 1.64 in the AAK group, and 1.87 ± 1.48 in the TCC group, while that at Week 16 were 2.48 ± 1.54 in the AAK group, and 2.78 ± 1.67 in the TCC group. Compared to Week 12, there was no statistical difference between either group during the follow‐up period at Week 16 (*p* = 0.344) (Table 3).

**TABLE 2:** Melanin index (MI).

MI	AAK			TCC			Mean difference (95% CI)	*p*
	Mean ± SD	Mean difference (95% CI)	*p*	Mean ± SD	Mean difference (95% CI)	*p*		
Baseline	286.89 ± 70.83	—	—	283.36 ± 73.97	—	—	—	—
Week 4	275.69 ± 74.37	−11.2 (−47.88, 25.48)	0.55	262.78 ± 84.45	−20.58 (−61.18, 20.02)	0.321	9.38 (−13.67, 32.44)	0.425
Week 8	269.32 ± 66.44	−17.57 (−54.25, 19.11)	0.348	245.58 ± 83.71	−37.78 (−78.38, 2.83)	0.068	20.21 (−2.85, 43.27)	0.086
Week 12	266.77 ± 67.75	−20.12 (−57.15, 16.91)	0.287	241.68 ± 73.26	−41.68 (−82.67, −0.69)	0.046	21.64 (−1.7, 44.99)	0.069
Week 16	296.42 ± 70.89	29.65 (−8.5, 67.79)*	0.128*	303.49 ± 70.69	61.81 (19.58, 104.04)*	0.004*	−32.16 (−89.07, 24.75)	0.268

Abbreviations: AAK, alpha‐arbutin 5% and kojic acid 2%; CI, confidence interval; TCC, triple combination cream.

* Compared with week 12.

**TABLE 3:** Modified Melasma Area Severity Index (mMASI).

mMASI	AAK			TCC			Mean difference (95% CI)	*p*
	Mean ± SD	Mean difference (95% CI)	*p*	Mean ± SD	Mean difference (95% CI)	*p*		
Baseline	2.83 ± 1.79	—	—	2.77 ± 1.7	—	—	—	—
Week 4	2.46 ± 1.75	−0.37 (−1.26, 0.52)	0.417	2.25 ± 1.69	−0.52 (−1.37, 0.34)	0.239	0.15 (−0.16, 0.46)	0.352
Week 8	2.28 ± 1.75	−0.55 (−1.44, 0.34)	0.228	1.99 ± 1.64	−0.78 (−1.63, 0.08)	0.076	0.23 (−0.08, 0.54)	0.147
Week 12	2.19 ± 1.64	−0.64 (−1.54, 0.25)	0.16	1.87 ± 1.48	−0.9 (−1.76, −0.03)	0.043	0.25 (−0.06, 0.56)	0.119
Week 16	2.48 ± 1.54	0.29 (−0.63, 1.22)*	0.534	2.78 ± 1.67	0.91 (0.02, 1.81)*	0.045*	−0.62 (−0.95, −0.3)	0.344

Abbreviations: AAK, alpha‐arbutin 5% and kojic acid 2%; CI, confidence interval; TCC, triple combination cream.

* Compared with Week 12.

**FIGURE 2:**
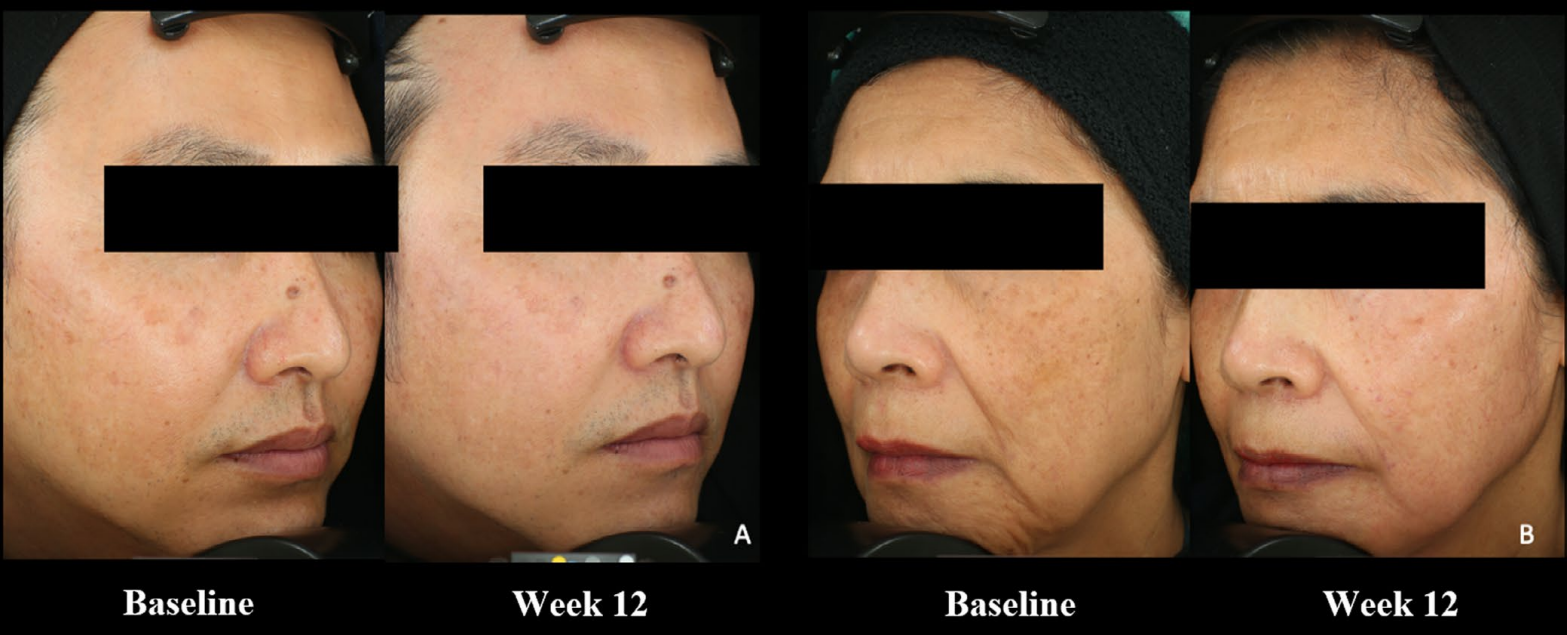
Clinical photography. (A) Alpha‐arbutin 5% and kojic acid 2% side and (B) triple combination cream side.

The example of clinical photography comparing at baseline and Week 12 was demonstrated in Figure 2. The overall PGA scores were significantly higher in the TCC groups over the study period: Week 4 (*p* = 0.002), Week 8 (*p* < 0.01), and Week 12 (*p* = 0.032) (Table 4). In brief, at Week 4, in the AAK group, 55.6% of the participants did not improve their scores, and 44.4% were slightly improved. In the TCC group, they were 33.3% (no improvement), 33.3% (slight improvement), 29.6% (fair), and 3.7% (good). At Week 8, in the AAK group, the scores were 29.6% (no improvement), 63% (slightly improved), and 7.4% (fair). In the TCC group, 18.5% of the participants did not improve, 22.2% (slight improvement), 40.7% (fair), 14.8% (good), and 3.7% (excellent). At Week 12, in the AAK group, 14.8% of the participants had no improvement, 51.9% (slightly improved), 18.5% (fair), and 11.1% (good). In the TCC group, 3.7% of the participants had no improvement, 33.3% (slight improvement), 37% (fair), 18.5% (good), and 3.7% (excellent). Furthermore, the overall patient satisfaction scores were not different between the AAK and TCC groups (*p* = 0.065) (Table 5). Most of participants with the AAK had moderate satisfaction (40.7%), 33.3% had slightly satisfaction, and 22.2% were very satisfied, whereas almost half of the participants with the TCC were very satisfied with the outcomes (44.44%), followed by moderate (37%), and slightly satisfied (14.8%).

**TABLE 4:** Physician global assessment (PGA) score.

PGA score	Week 4		Week 8		Week 12	
	AAK (%)	TCC (%)	AAK (%)	TCC (%)	AAK (%)	TCC (%)
0 (No improvement)	15 (55.6)	9 (33.3)	8 (29.6)	5 (18.5)	4 (14.8)	1 (3.7)
1 (Slight improvement)	12 (44.4)	9 (33.3)	17 (63)	6 (22.2)	14 (51.9)	9 (33.3)
2 (Fair)	0	8 (29.6)	2 (7.4)	11 (40.7)	5 (18.5)	10 (37)
3 (Good)	0	1 (3.7)	0	4 (14.8)	3 (11.1)	5 (18.5)
4 (Excellent)	0	0	0	1 (3.7)	0	1 (3.7)
*p*	0.002		<0.001		0.032	

Abbreviations: AAK, alpha‐arbutin 5% and kojic acid 2%; TCC, triple combination cream.

**TABLE 5:** Patient satisfaction score.

Patient satisfaction	Week 12	
	AAK (%)	TCC (%)
Very satisfied	6 (22.2%)	12 (44.4%)
Moderately satisfied	11 (40.7%)	10 (37%)
Slightly satisfied	9 (33.3%)	4 (14.8%)
*p*	0.065	

Abbreviations: AAK, alpha‐arbutin 5% and kojic acid 2%; TCC, triple combination cream.

The observed side effects among participants in both groups as early as Week 4 were erythema, dryness, stinging, and itching. None of the participants in both groups experienced telangiectasia, atrophy, or hypopigmentation (Table 6). Erythema was more significant in the TCC group, which was presented approximately 33% at Week 4, while there were 11.1% of participants in the AAK group who experienced this event (*p* = 0.024). At Week 8, erythema was only found to be mild in both groups but was more apparent in the TCC group (40.7% vs. 11.1%) (*p* = 0.011). By Week 12, erythema could still be observed in the TCC group (11.1%) whereas this was no longer detected in the AAK group (*p* = 0.083). For dryness, the TCC showed a higher trend compared with the AAK throughout the study period though there was no statistically significant difference (*p* = 0.317). Approximately 15% of the TCC group had skin dryness at Week 4 (mild: 11.1% and moderate: 3.7%) and Week 8 (mild: 14.8%), while that of the AAK were approximately 7%; mild (3.7%) and moderate (3.7%) at Week 4, and mild (7.4%) at Week 8. At Week 12, only one participant treated with the TCC experienced dryness (3.7%), whereas none with the AAK observed such an event.

**TABLE 6:** Side effects.

	Week 4		Week 8		Week 12		Week 16	
	AAK (%)	TCC (%)	AAK (%)	TCC (%)	AAK (%)	TCC (%)	AAK (%)	TCC (%)
Erythema								
0 = none	24 (88.9%)	18 (66.7%)	24 (88.9%)	16 (59.3%)	26 (96.3%)	23 (85.2%)	23 (85.2%)	23 (85.2%)
1 = mild	3 (11.1%)	6 (22.2%)	3 (11.1%)	11 (40.7%)	0 (0%)	3 (11.1%)	1 (3.7%)	1 (3.7%)
2 = moderate	0 (0%)	3 (11.1%)	0 (0%)	0 (0%)	0 (0%)	0 (0%)	0 (0%)	0 (0%)
3 = severe	0 (0%)	0 (0%)	0 (0%)	0 (0%)	0 (0%)	0 (0%)	0 (0%)	0 (0%)
*p*	0.024		0.011		0.083		1.000	
Dryness								
0 = none	25 (92.6%)	23 (85.2%)	25 (92.6%)	23 (85.2%)	26 (96.3%)	25 (92.6%)	24 (88.9%)	24 (88.9%)
1 = mild	1 (3.7%)	3 (11.1%)	2 (7.4%)	4 (14.8%)	0 (0%)	1 (3.7%)	0 (0%)	0 (0%)
2 = moderate	1 (3.7%)	1 (3.7%)	0 (0%)	0 (0%)	0 (0%)	0 (0%)	0 (0%)	0 (0%)
3 = severe	0 (0%)	0 (0%)	0 (0%)	0 (0%)	0 (0%)	0 (0%)	0 (0%)	0 (0%)
*p*	0.317		0.317		0.317		1.000	
Stinging								
0 = none	24 (88.9%)	19 (70.4%)	27 (100%)	21 (77.8%)	26 (96.3%)	25 (92.6%)	24 (88.9%)	24 (88.9%)
1 = mild	3 (11.1%)	6 (22.2%)	0 (0%)	5 (18.5%)	0 (0%)	0 (0%)	0 (0%)	0 (0%)
2 = moderate	0 (0%)	1 (3.7%)	0 (0%)	0 (0%)	0 (0%)	1 (3.7%)	0 (0%)	0 (0%)
3 = severe	0 (0%)	0 (0%)	0 (0%)	0 (0%)	0 (0%)	0 (0%)	0 (0%)	0 (0%)
*p*	0.059		0.025		0.317		1.000	
Itching								
0 = none	25 (92.6%)	24 (88.9%)	27 (100%)	27 (100%)	26 (96.3%)	25 (92.6%)	24 (88.9%)	24 (88.9%)
1 = mild	2 (7.4%)	3 (11.1%)	0 (0%)	0 (0%)	0 (0%)	1 (3.7%)	0 (0%)	0 (0%)
2 = moderate	0 (0%)	0 (0%)	0 (0%)	0 (0%)	0 (0%)	0 (0%)	0 (0%)	0 (0%)
3 = severe	0 (0%)	0 (0%)	0 (0%)	0 (0%)	0 (0%)	0 (0%)	0 (0%)	0 (0%)
*p*	0.564		1.000		0.317		1.000	

Abbreviations: AAK, alpha‐arbutin 5% and kojic acid 2%; TCC, triple combination cream.

Regarding stinging sensation, at Week 4, participants in the TCC group had mild (22.2%) and moderate stinging (3.7%), while in the AAK group, only mild stinging was found (11.1%) (*p* = 0.059). Stinging was reported exclusively in the TCC group at Week 8 (mild: 18.5%) (*p* = 0.025) and Week 12 (moderate: 3.7%) (*p* = 0.317). In addition, itching was reported as mild at Week 4 (11.1% in the TCC group, 7.4% in the AAK group) (*p* = 0.564). No participants reported itching at Week 8, but there was one in the TCC group who experienced mild itching at Week 12 (3.7%) (*p* = 0.317).

## 4 Discussion

To date, the combination of arbutin and kojic acid has shown some clinical benefits in melasma treatment. It has been proven that this was as effective as hydroquinone monotherapy [9]. To the best of our knowledge, this is the first split‐face randomized study that investigate the effects of combination cream containing AAK compared with the standard treatment, TCC.

Our study revealed non‐significant differences of MI and mMASI scores among the participants received either the AAK or TCC. By Week 12, when the interventions were terminated, both groups showed improvement of melasma from baseline. Interestingly, only the participants with TCC showed higher severity of recurrence at Week 16. Similarly, clinical improvement assessed by the PGA showed improvement in both groups; however, the overall PGA score was significantly higher in the TCC compared with the AAK group. Although the improvement of PGA score was seen in the AAK by the end of Week 12, the number of the AAK who were graded as “Fair” and “Good” was less than those of the TCC, while none of the AAK sides achieved the level of “Excellent.” Non‐significant difference of patient satisfaction score was observed between both groups, but nonetheless, erythema and stinging were significantly higher in the TCC group.

Regarding the outcomes, the results estimated by objective assessments indicated that the AAK was comparable to the TCC since the significant difference between groups was not observed when evaluated by MI. However, the efficacy of the TCC seemed to be superior to that of the AAK only when assessed by subjective evaluations such as PGA and patients satisfaction scores. Yet delay response needs to be aware. It should be highlighted that erythema was more frequently observed among those who received the TCC, while this was significantly less in the AAK. This was consistent with the results in the previous studies [13, 14] demonstrated that erythema was the most common side effect when using such interventions. As erythema was mainly observed in the TCC group, hydroquinone and tretinoin were responsible for this phenomenon. Interestingly, the degree of erythema was transient and tolerable and thus did not lead to treatment discontinuation. This side effect was uncommon after continuous use of the TCC and AAK. Stinging and itching were frequently detected in both groups during the first 4 weeks. After that, these were only observed among the participants who received the TCC throughout the study period. The participants with the TCC showed higher trend of dryness during their application. Unlike previous reports, other side effects such as hypopigmentation, atrophy, or telangiectasia were not detected in both the TCC and AAK groups [5, 13, 14]. Melasma recurred after cessation of treatment in both groups. Though greater reduction of MI and mMASI during the application period, the TTC carried a higher risk of recurrence as our results showed that the severity of recurrent melasma was increased exclusively in the TCC group. This rebound effect at Week 12 appeared to be statistically significant when compared to baseline MI and mMASI at initial of the study. The increased value of MI and mMASI may be partly attributed to post inflammatory hyperpigmentation following irritation [7].

The results of this study were consistent with the study by Sardesai et al. [15] demonstrating that the modified Kligman's formula was more effective than arbutin and kojic acid in reducing centrofacial melasma, on the other hand, had higher side effects. Short‐term side effects of the TCC were reversible but skin atrophy, known as long‐term complication, was rare [16]. It was evident that the prevalence of skin atrophy was low even when TCC was continued for 12 months [17]. Since none evidence of that after 12 months is available, increasing risk of atrophy after prolonged application for years is still questionable. Another issue worth mentioning regarding side effects is exogenous ochronosis. It was recommended that hydroquinone at a concentration of ≤1% was considered to be safe [18]. Since melasma is a chronic and recalcitrant skin condition, prolonged exposure to hydroquinone in the TCC, which is generally higher than 2%, should be avoided to reduce the risk of ochronosis and atrophy. The results of the current study revealed that the AAK showed some clinical benefits for melasma which were consistent with previous studies. Kojic acid at a concentration of 2% was beneficial in melasma patients who were not responsive to hydroquinone [19, 20, 21]. While irritation and redness may be observed after treating with this, serious side effects were uncommon [12]. It is noteworthy that arbutin could be another fascinating substitute in melasma treatment as it is less toxic than hydroquinone [22]. We believed that adding the AAK to the standard therapy may lead to synergistic effects, then promoting treatment outcomes. Besides, the AAK can also be initiated as a maintenance treatment, after termination of the TCC, due to its satisfactory safety profile.

The main limitation of this study is, first, the small number of sample sizes (*n* = 30). A larger sample size would be desirable to provide more accurate values with smaller margins of error and lower standards of deviation, thus increasing reliability and validity of the findings. Upon increasing the number of subjects, statistical differences between the two groups may become noticeable. Second, the follow‐up period in this study was generally short, thus we could not capture any delayed undesirable consequences that might have occurred beyond this period. As such, the extended follow‐up duration is recommended to observe long‐term complications among participants receiving both TCC and AAK.

To sum up, our results indicated that the clinical efficacy of the AAK could be equivalent to the TCC when assessed by objective assessment such as MI, while incomparable to the TCC when evaluated subjectively. It should be highlighted that despite the relatively better outcome after the TCC treatment, higher chance of rebound could be expected after the TCC discontinuation. In contrast, the AAK had lower risk of recurrence and less side effects. As clinical effect of the TCC was not sustained after treatment cessation, the AAK may be worthwhile as a maintenance treatment for melasma.

## 5 Conclusions

Our findings shed light on the benefits of the AAK that may be suitable as the treatment option in sensitive skin patients with melasma or in maintenance treatment due to less chance of recurrence and minimal side effects.

## Author Contributions

P.T. and B.C. conceptualized the design and execution of the study. P.T. and A.S. gathered data and appraised the articles. P.T., A.S., and B.C. performed the analysis, data interpretation, and drafted the manuscript. All authors discussed the results and contributed to the final version of the manuscript.

## Ethics Statement

The authors confirm that the ethical policies of the journal, as noted on the journal's author guidelines page, have been adhered to and the appropriate ethical review committee (Ethics committee of the Institute of Dermatology, Bangkok, Thailand) approval with the study code, IRB 029/2564 on 29 September 2021 has been received.

## Consent

The authors certify that they have obtained all appropriate patient consent.

## Conflicts of Interest

The authors declare no conflicts of interest.

## Data Availability

The data that support the findings of this study are available from the corresponding author upon reasonable request.
